# Antimicrobial Resistance and β-Lactamase Production in Clinically Significant Gram-Negative Bacteria Isolated from Hospital and Municipal Wastewater

**DOI:** 10.3390/antibiotics12040653

**Published:** 2023-03-26

**Authors:** Mohammad Irfan, Alhomidi Almotiri, Zeyad Abdullah AlZeyadi

**Affiliations:** Department of Clinical Laboratory Sciences, College of Applied Medical Sciences, Shaqra University, Shaqra 11961, Saudi Arabia; hsalmutiri@su.edu.sa (A.A.); zalzeyadi@su.edu.sa (Z.A.A.)

**Keywords:** wastewater, sewage, ESBL, carbapenemase, antibiotic resistance, antibiotic-resistant bacteria

## Abstract

Hospital and municipal wastewater contribute to the spread of antibiotic-resistant bacteria and genes in the environment. This study aimed to examine the antibiotic resistance and β-lactamase production in clinically significant Gram-negative bacteria isolated from hospital and municipal wastewater. The susceptibility of bacteria to antibiotics was tested using the disk diffusion method, and the presence of extended-spectrum β-lactamases (ESBL) and carbapenemases was determined using an enzyme inhibitor and standard multiplex PCR. Analysis of antimicrobial resistance of total bacterial strains (*n* = 23) revealed that most of them were resistant to cefotaxime (69.56%), imipenem (43.47%), meropenem (47.82%) and amoxicillin-clavulanate (43.47%), gentamicin (39.13%), cefepime and ciprofloxacin (34.78%), trimethoprim-sulfamethoxazole (30.43%). A total of 8 of 11 phenotypically confirmed isolates were found to have ESBL genes. The *bla*_TEM_ gene was present in 2 of the isolates, while the *bla*_SHV_ gene was found in 2 of the isolates. Furthermore, the *bla*_CTX-M_ gene was found in 3 of the isolates. In one isolate, both the *bla*_TEM_ and *bla*_SHV_ genes were identified. Furthermore, of the 9 isolates that have been phenotypically confirmed to have carbapenemase, 3 were confirmed by PCR. Specifically, 2 isolates have the *bla*_OXA-48_ type gene and 1 have the *bla*_NDM-1_ gene. In conclusion, our investigation shows that there is a significant rate of bacteria that produce ESBL and carbapenemase, which can promote the spread of bacterial resistance. Identifying ESBL and carbapenemase production genes in wastewater samples and their resistance patterns can provide valuable data and guide the development of pathogen management strategies that could potentially help reduce the occurrence of multidrug resistance.

## 1. Introduction

Hospital wastewater is considered potentially hazardous due to the presence of pharmaceutical residues, radioisotopes, and microbes, which could pose risks to human and environmental health. Antibiotics used in hospitals can end up in wastewater through patient urine and feces, selecting multidrug resistant (MDR) bacteria to thrive. This is because the human body is unable to fully metabolize some of the active ingredients in these drugs [1]. The sewer system in metropolitan areas transports antibiotic residues and resistant bacteria from people and/or animals to discharge sites, such as wastewater treatment facilities [2]. The MDR bacteria that produce hydrolyzing enzymes are found regularly in hospitals but have also been found in environmental sources such as rivers, seawater, and wastewater from both urban and hospital sources [3,4,5,6]. Studies have found that municipal wastewater treatment plants can harbor MDR bacteria that can spread to the environment [7,8]. The prevalence of antibiotic-resistant bacteria (ARB) varies over time and between hospitals and wastewater treatment plants (WWTP) in different locations [9]. The spread of extended-spectrum β-lactamases (ESBL) has become an important factor in the emergence of gram-negative MDR bacteria in hospitals, identified in Europe and then worldwide [10]. The β-lactam class of antibiotics is the most commonly prescribed group of antibacterial agents, accounting for approximately 70% of prescriptions, due to their ability to effectively target a wide range of bacteria [11]. These antibiotics are commonly used to treat severe infections, but their effectiveness has been compromised by the emergence of ESBL and carbapenemase, which have a negative impact on their clinical use [12].

The World Health Organization (WHO) has identified carbapenemase-producing *Enterobacteriaceae* (CRE) as a critical threat to human health and has included it in its list of priority diseases for which new treatments are urgently needed. CRE has become a major concern in recent years due to its resistance to antibiotics and the proliferation of antibiotic-resistant pathogens, which poses a significant challenge for Saudi Arabia [13]. The transmission of antibiotic-resistant strains of *Enterobacteriaceae* is a significant concern, and the large volume of human movement (pilgrim, tourism, work) within and outside the Gulf region is a major risk factor for their spread. These strains have been documented in several studies in various regions of Saudi Arabia [14,15,16,17,18,19]. There is a lack of data on the prevalence and patterns of antimicrobial resistant bacteria in hospital and municipal wastewater in Saudi Arabia. Therefore, we conducted this study to determine the resistance patterns of clinically significant isolates to various antibiotics and to investigate the presence of ESBL and carbapenemase-producing bacteria. The study also aimed to determine the presence of specific ESBL and carbapenemase genes in isolated organisms.

## 2. Results

### 2.1. Bacterial Isolation

In this study, 23 bacterial isolates from MacConkey agar containing 2 µg of meropenem were selected (as shown in Table 1). Of these isolates, 7 were obtained from hospital wastewater samples, 14 were obtained from municipal wastewater samples, and 2 were obtained from municipal treated wastewater samples. No isolates were obtained from treated wastewater samples collected from the hospital.

### 2.2. Identification of Isolates

Of 23 isolates, 13 were found to belong to the *Enterobacteriaceae* family, including 3 *E. coli*, 4 *Klebsiella* spp., 3 *Enterobacter* spp., 2 *Citrobacter* spp. and 1 *Proteus* spp. The remaining 11 strains were identified as non-*Enterobacteriaceae*, including 5 *Acinetobacter* spp. and 5 *Pseudomonas* spp. Of the total of seven isolates from hospital wastewater, the most predominant taxa were *Acinetobacter* spp. followed by *Klebsiella* spp., *Enterobacter* spp. and *Pseudomonas* spp. In the wastewater and treated wastewater samples collected from the municipality, we identified 16 strains of bacteria. The most common strains were *Pseudomonas* spp. followed by *E. coli*, *Klebsiella* spp., *Acinetobacter* spp., *Enterobacter* spp., *Citrobacter* spp., and *Proteus* spp. (Table S1, supplementary materials).

### 2.3. Determination of the Antimicrobial Susceptibility Pattern of the Isolates

In our study, the antibiotic susceptibility patterns of the *Enterobacteriaceae* strains were as follows: *Enterobacter* spp. (*n* = 3) Isolated from the hospital and municipal wastewater and treated wastewater were found resistant to amoxicillin-clavulanate (66.66%), cefotaxime (100%), imipenem (33.33%), meropenem (33.33%), gentamicin (66.66%), ciprofloxacin (33.33%) and trimethoprim-sulfamethoxazole (33.33%). *Klebsiella* spp. (*n* = 4) isolated from hospital and municipal wastewater showed resistance to amoxicillin-clavulanate (50%), cefotaxime (75%), cefepime (25%), imipenem (75%), meropenem (50%), ciprofloxacin (25%), gentamicin (25%) and trimethoprim-sulfamethoxazole (50%). *E. coli* spp. (*n* = 3) isolated from municipal wastewater showed resistance to ceftazidime (33.33%), cefotaxime (100%), imipenem (33.33%), meropenem (33.33%), ciprofloxacin (66.66%), gentamicin (33.33%) and trimethoprim-sulfamethoxazole (33.33%). *Citrobacter* spp. (*n* = 2) isolated from municipal wastewater were found to be resistant to amoxicillin-clavulanate (100%), ceftazidime (50%), cefepime (50%), cefotaxime (50%), imipenem (50%), meropenem (50%), ciprofloxacin (50%) and trimethoprim-sulfamethoxazole (50%). *Proteus* spp. (*n* = 1) isolated from municipal wastewater were resistant to amoxicillin-clavulanate (100%), ceftazidime (100%), cefotaxime (100%), cefepime (100%), meropenem (100%) and trimethoprim-sulfamethoxazole (100%). Analysis of 13 *Enterobacteriaceae* strains for antimicrobial resistance (AMR) showed that most of them were resistant to cefotaxime (84.61%), followed by amoxicillin-clavulanate (53.84%), imipenem and meropenem (46.15% each), trimethoprim-sulfamethoxazole (38.46%) and ciprofloxacin (30.76%).

The antibiotic susceptibility pattern of strains other than *Enterobacteriaceae* in our study was recorded as *Acinetobacter* spp. (*n* = 5) isolated from hospital and municipal wastewater were found to be resistant to cefotaxime (80%), cefepime (60%), imipenem (40%), meropenem (40%), ciprofloxacin (20%), gentamicin (20%) and trimethoprim-sulfamethoxazole (40%). *Pseudomonas* spp. (*n* = 5) isolated from hospital and municipal wastewater and from treated municipal wastewater were resistant to ceftazidime (40%), cefotaxime (20%), cefepime (40%), imipenem (40%), meropenem (60%), ciprofloxacin (60%) and gentamicin (80%). The AMR patterns of 10 strains of *non-Enterobacteriaceae* were also examined, and it was found that most showed resistance to cefotaxime, cefepime, Gentamicin, and Meropenem (50.00%), Imipenem and ciprofloxacin (40%).

An analysis of 23 bacterial strains found that most were resistant to cefotaxime (69.56%), followed by meropenem (47.82%), imipenem and amoxicillin-clavulanate (43.47%), gentamicin (39.13%), cefepime and ciprofloxacin (34.78%), and trimethoprim-sulfamethoxazole (30.43%). No bacteria were found to be resistant to colistin (Table S2, supplementary materials).

### 2.4. Screening for β-Lactamases

Of the 23 bacterial strains isolated from municipal and hospital wastewater and treated wastewater, 18 were resistant to cefotaxime/ceftazidime or both antibiotics. The 18 potential ESBL strains were further tested for confirmation of ESBL. 11 were confirmed to be producers of ESBL belonging to species *Acinetobacter, Enterobacter, Pseudomonas, E. coli, Citrobacter,* and *Proteus.* 13 isolates were found to be resistant to imipenem and/or meropenem; out of which 9 isolates were positive for Modified Hodge Test (MHT). The isolates were belonging to *Klebsiella, Citrobacter, Enterobacter, Acinetobacter* and *Pseudomonas* species (Figure S1 and S2, supplementary materials).

### 2.5. Detection of Resistance Genes by PCR

Of the 11 phenotypically confirmed ESBL isolates, 8 carried ESBL genes. The *bla*_TEM_ gene was identified in a total of 2 strains, mainly harbored in the *Pseudomonas and Enterobacter* species. Furthermore, the *bla*_SHV_ gene was identified in 2 strains mainly harbored by *Citrobacter and Proteus* species. The *bla*_CTX-M_ gene was identified in 3 strains mainly harbored by *Acinetobacter* species. Of the total PCR-positive ESBL isolates, the coexistence of two different genes, that is, *bla*_TEM_ and *bla*_SHV_, in a single isolate was revealed in a *E. coli.* The most common carbapenemase encoding gene found in this study was *bla*_OXA-48_ and *bla*_NDM-1_. It was present in 3 of the 9 phenotypically confirmed carbapenemase producing isolates. 2 of *Klebsiella* species were found to harbor *bla*_OXA-48_. These isolates came from the hospital and municipal wastewater. The second prevalent carbapenemase encoding gene found was *bla*_NDM-1_, which was present in 1 of *Acinetobacter* spp. which was isolated from municipal wastewater (Figures S3 and S4 Supplementary Materials).

## 3. Discussion

The overuse and abuse of antimicrobials in human and veterinary medicine, as well as environmental contamination, have contributed significantly to the emergence of AMR as a major threat to public health. The most common causes of AMR in healthcare facilities are the inappropriate use of antimicrobials and an inadequate infection control program. It has been recognized that aquatic environments can serve as a means of transmission of infection, and this is often related to the discharge of wastewater effluents from medical facilities, animal breeding farms, and sewer systems [20]. Despite this, there is a lack of conclusive information on the conditions or mechanisms that contribute to the development of drug-resistant strains in individuals [21]. Recent research has shown that bacteria and their genetic material can be easily transferred between humans, animals, and the environment [22,23,24].

In our study, the most predominant species identified in hospital wastewater were *Klebsiella* spp., *Acinetobacter* spp. *Enterobacter* spp. and *Pseudomonas* spp. The strains identified in municipal wastewater and treated wastewater samples were dominated by *Pseudomonas* spp., *E. coli*, *Enterobacter* spp., *Citrobacter* spp., *Klebsiella* spp., *Acinetobacter* spp. and *Proteus* species at the lowest abundance. Similarly, a study by Khaled et al. characterized bacterial species isolated from domestic wastewater treatment plants in Jazan, KSA, describing several enteric and non-enteric Gram-negative strains [25]. A study by Röderová et al. also found human and environmental bacteria in wastewater from hospitals and urban wastewater treatment plants [26].

We found that isolates from hospital wastewater, as well as municipal wastewater and treated wastewater, showed resistance to various antibiotics. The results of our analysis of the AMR of *Enterobacteriaceae* strains showed that most of them were resistant to cefotaxime (84.61%), amoxicillin-clavulanate (53.84%), imipenem and meropenem (46.15%) and trimethoprim-sulfamethoxazole (38.46%). Ciprofloxacin had a lower resistance level of 30.76%. The results of our study are consistent with previous research on resistance patterns of *Enterobacteriaceae* strains, such as a study conducted in Germany that found a high level of resistance to cefotaxime (89%), ceftazidime (95%) and ciprofloxacin (53%) [27]. The resistance pattern of the *non-Enterobacteriaceae* strains revealed that most of them showed resistance to cefotaxime, cefepime, Gentamicin and Meropenem (50.00%), Imipenem and ciprofloxacin (40%), and many studies have found carbapenem-resistant *Acinetobacter* spp. in hospital wastewater [28,29,30], but resistance to carbapenems has rarely been studied in isolates obtained from municipal wastewater [31].

A major concern for global public health is the rapid increase in the incidence of *Enterobacteriaceae* producing ESBL and its subsequent spread to the general population. The frequency of ESBL production was highest among *Acinetobacter*, followed by *Enterobacter, Pseudomonas, E. coli*, *Citrobacter and Proteus*. A study by Bréchet et al. found a high prevalence of *Enterobacteriaceae* producing ESBL in hospital wastewater [32]. Furthermore, global studies have found significant variations in the prevalence and percentage of strains that produce ESBL between different nations [33]. It appears that the production of ESBL by *Enterobacteriaceae* and non-*Enterobacteriaceae* strains may restrict the treatment options for infections caused by this group of bacteria. Therefore, the bacteria that produce ESBL pose a significant problem that requires proactive efforts to prevent their occurrence.

In our study, carbapenemase producers were phenotypically identified among *Klebsiella, Citrobacter, Enterobacter, Acinetobacter, and Pseudomonas species.* Two separate phenotypic studies conducted in Mecca found that 48.4% and 38% of the samples were positive for carbapenemase production in *Klebsiella pneumoniae* [34,35]. Wastewater serves as a reservoir of antibiotic resistant bacteria that reflects the composition of bacterial populations carried out by the general population, and wastewater is a hotspot for the exchange of resistant genes among bacteria [36]. Isolates that were found to produce ESBL or carbapenemase by any phenotypic method were further characterized by molecular analysis. The *bla*_TEM_-type gene was detected in 2 strains of *Pseudomonas* spp. and *Enterobacter* spp. isolated from municipal wastewater and treated wastewater, respectively. The *bla*_SHV_-type gene was also found in 2 strains, mainly in *Citrobacter* and *Proteus* spp. isolates from municipal wastewater. The *bla*_CTX-M_ type gene was identified in 3 strains, mainly in *Acinetobacter* spp. isolates from the hospital and municipal wastewater. The prevalence of the *bla*_TEM_ gene in this study is similar to the findings of a study conducted in Portugal, where the most commonly identified genes were *bla*_TEM_ (24.1%) and *bla*_CTX-M_ (5.6%) [37]. In a separate study, the *bla*_TEM_ gene was found to be the most prevalent in wastewater samples collected during biological treatment, including treated wastewater. However, in another study, the *bla*_SHV_ gene was the least prevalent among the genes tested [38]. A recent study revealed that a small percentage of ESBL genes were found in isolates obtained from wastewater effluent. Among these samples, 9.2% carried the *bla*_TEM_ gene, 1.4% carried the *bla*_SHV-12_ gene, 0.2% carried the *bla*_CTX-M-1_ gene, and 1% carried the *bla*_CTX-M-15_ gene [39]. In WWTP, the genes *bla*_CTX-M_, *bla*_TEM_, *bla*_SHV_, and *bla*_OXA_ have been identified in multiple species within the *Enterobacteriaceae* species group. Our study identified a combination of *bla*_TEM_ and *bla*_SHV_ genes in 1 *E. coli* spp. isolated from municipal wastewater. In other studies, *bla*_SHV_ and/or *bla*_TEM_ were also frequently found [40,41,42]. Furthermore, we found that the genes *bla*_OXA-48_ and *bla*_NDM-1_ were prevalent among carbapenemase genes. Specifically, the *bla*_OXA-48_ gene was the most identified in *Klebsiella* spp. isolated from hospital and municipal wastewater. The presence of *bla*_OXA-48_ has also been detected in Saudi Arabian clinical samples from Saudi Arabia [43]. Studies also reported the prevalence of *bla*_OXA-48_ in wastewater [44,45]. Meanwhile, the *bla*_NDM-1_ gene was detected in *Acinetobacter* spp. recovered from municipal wastewater. Our results are consistent with those of previous studies showing that the *bla*_NDM-1_ gene has been found in a wide variety of species and is spreading rapidly in various environments [28,46,47]. In this study, none of the isolates tested positive for carbapenemase encoding genes of type *bla*_IMP_, *bla*_VIM_ or *bla*_KPC_.

Our research had some limitations, including the fact that we only tested β lactamase genes in bacteria that were phenotypically confirmed, the list of resistance testing primers did not cover everything, and we did not test the mechanism of resistance to carbapenems other than the production of carbapenemase. Resistance genes could be present in bacteria that do not show resistance phenotypically. The only way to overcome this is to perform whole genome sequencing for all isolates. Furthermore, our sample size was relatively small, so future studies with more samples from various sources will provide a more comprehensive understanding of pathogens and β-lactam genes. Another limitation was that our study only looked at a hospital and a municipal wastewater treatment plant in a specific location, so the results may not apply to other locations.

## 4. Materials and Methods

### 4.1. Sample Collection

Six samples, consisting of three wastewater samples and three treated wastewater samples, were collected from a hospital and municipal wastewater treatment plant in the central region of Saudi Arabia between October 2021 and December 2021. The samples were collected between 8 am and 11 am and placed in 1-L plastic containers that had been sterilized with 70% alcohol and rinsed with deionized water. After collection, the samples were transported in an insulated box with ice packs to the laboratory for processing. All samples were stored at a temperature of 4 °C and analyzed within 24 h after collection.

### 4.2. Bacterial Isolates

10 mL of wastewater and 100 mL of treated wastewater were centrifuged at 5000× *g* for 10 min. The samples were then serially diluted with normal sterile saline (10^−1^, 10^−2^, 10^−3^) and 100 μL of each aliquot was spread on MacConkey agar with 2 μg/mL of meropenem added. The plates were incubated overnight at a temperature of 35 ± 2 °C. To further purify the bacteria, colonies with distinctive color and characteristics were randomly selected and subcultured on MacConkey agar containing 2 μg of meropenem. The isolates were then stored in TSA + 10% glycerol stock at a temperature of −80 °C for further analysis. The identifications of the isolates were confirmed by Gram staining and then further determined by biochemical analysis, as detailed by Mahan et al. [48].

### 4.3. Antimicrobial Sensitivity

The antibiotic sensitivity test (AST) was performed using the disk diffusion method as per the recommendations of the Clinical Laboratory Standard Institute (CLSI) [49]. The disk diffusion test was carried out using the Kirby-Bauer technique with cefotaxime (CTX, 30 μg), cefepime (FEP, 5 μg), imipenem (IMP, 10 µg), meropenem (MEM, 10 μg), ciprofloxacin (CIP, 5 μg), gentamicin (GEN, 10 μg), trimethoprim/sulfamethoxazole (SXT, 23.75 μg + 1.25 μg), ceftazidime (CAZ, 30 μg), amoxicillin-clavulanate (AMC, 30 µg), and colistin (CST) (30 µg). All antibiotics were obtained from Oxide Pvt. Ltd. The quality control process included the use of strains from the American Type Culture Collection (ATCC) *Escherichia coli* (*E. coli)* 25,922 and *P. aeruginosa* 27,953.

### 4.4. Phenotypic Detection of β-Lactamases

A double disc diffusion test [49] was carried out to verify the presence of ESBL, discs containing ceftazidime (30 μg), cefotaxime (30 μg), and a combination of clavulanic acid (30 μg)/10 μg) were used. The disks were placed at the appropriate distance on MHA plates that had been inoculated with a bacterial suspension of 0.5 McFarland turbidity standards. The plates were left to incubate for 18–20 h at 37 °C. The isolates were determined to be producers of ESBL if the difference in the zone of inhibition of the drug and inhibitor was at least 5 mm compared to cephalosporin alone.

Modified Hodge Test [49] was performed to determine carbapenemase production according to CLSI standards. A suspension of *E. coli* ATCC 25,922 was adjusted to the 0.5 McFarland standard and swabbed on MHA plates. After drying, a disc containing 10 μg of meropenem was left in the middle of the plate and then the isolates were spread in a thin line from the edge of the disc that extended to the edge of the plate. The plates were incubated overnight at 37 °C. A distorted inhibitory zone in the shape of a clover leaf on the meropenem disc around the growth of *E. coli* ATCC 25,922 indicated a positive result.

### 4.5. Analysis of Gene Molecules of ESBL and Carbapenemase

#### 4.5.1. DNA Extraction

Fresh colonies of all phenotypically verified ESBL and carbapenemase isolates were processed for DNA extraction using the boiling method [50]. Briefly, a suspension was made by suspending 2–4 fresh colonies of each isolate in 500 µL of nuclease-free distilled water. The suspension was heated to 95 °C for 10 min, followed by cooling and then centrifugation at 10,000 rpm for 10 min. Finally, 150 µL of the supernatant was stored at −20 °C and used as a template for subsequent amplification.

#### 4.5.2. Detection of Genes Encoding ESBL and Carbapenemase

The multiplex polymerase chain reaction (PCR) was performed in two separate reactions using the BIO-RAD T100 thermal cycler (Applied Biosystems, Waltham, MA, USA). In the first reaction, the genes *bla*_CTX-M_, *bla*_TEM,_ and *bla*_SHV_ were multiplexed in a single tube to detect ESBL. In the second reaction, the genes *bla*_IMP_, *bla*_VIM_, *bla*_KPC,_ *bla*_NDM_, and *bla*_OXA-48_ were multiplexed to detect carbapenemase. The PCR procedures were performed in a total volume of 20 µL, including 4 µL of 5× FIREPol^®^ Master Mix (Solis Biodyne, Tartu, Estonia), 0.2 µL of each primer, 1 µL of DNA template and 14.6 µL of nuclease-free water. The amplification cycle consisted of initial denaturation at 95 °C for 3 min, followed by 30 cycles of denaturation at 95 °C for 15 s, annealing at the temperature specified in Table 2 for 30 s, extension at 72 °C for 1 min, and final extension at 72 °C for 5 min. The PCR results were analyzed by gel electrophoresis on 1.5% agarose gel. The size of the amplicon was calculated compared to the 100 bp ladder marker (Solis Biodyne, Tartu, Estonia).

## 5. Conclusions and Future Perspectives

Our data indicate that that hospital and municipal wastewater contained a range of resistant bacteria dominated by *Klebsiella, Acinetobacter, Enterobacter, and Pseudomonas* species. These isolates showed resistance to various antibiotics, particularly cefotaxime, amoxicillin-clavulanate, imipenem, meropenem, and trimethoprim-sulfamethoxazole. Furthermore, Enterobacteriaceae and non-Enterobacteriaceae strains producing ESBL and carbapenemase were also identified. To address this issue, it is important to identify the genes responsible for producing ESBL and carbapenemase in nonclinical samples, such as wastewater. So, we can gain valuable information to help develop effective strategies for managing pathogens, which can help reduce the occurrence of multidrug resistance. Additionally, there is a practice of reusing wastewater for agricultural purposes, and the potential use of wastewater sludge for other applications. Given the high levels of resistant bacteria found in wastewater, we must take additional measures to remove these bacteria before releasing the water back into the environment or reusing it for other purposes. Therefore, it is essential to regularly evaluate wastewater systems in hospitals and municipalities. Furthermore, more research is needed to understand the risks associated with wastewater disposal and recycling. We must assess the extent of ecosystem pollution that occurs because of these practices. By doing so, we can develop more effective methods to manage wastewater and ensure that it does not contribute to the spread of antibiotic resistance in the environment.

## Figures and Tables

**Table 1:** List of bacteria isolated.

	Bacteria Isolated/Number of Isolates			
	Hospital (Wastewater)	Hospital (Treated Wastewater)	Municipal (Wastewater)	Municipal (Treated Wastewater)
*Enterobacteriaceae*	*Klebsiella* spp. (2)	No growth	*E. coli* (3)	*Enterobacter* spp. (1)
	*Enterobacter* spp. (1)		*Klebsiella* spp. (2)	
			*Enterobacter* spp. (1)	
			*Citrobacter* spp. (2)	
			*Proteus* spp. (1)	
*Non-Enterobacteriaceae*	*Acinetobacter* spp. (3)	No growth	*Acinetobacter* spp. (2)	*Pseudomonas* spp. (1)
	*Pseudomonas* spp. (1)		*Pseudomonas* spp. (3)	

**Table 2:** List of primers used.

Targeted Genes	Nucleotide Sequence (5′to 3′)	Amplicon Size (bp)	Annealing Temp	References
*bla*_CTX-M_	Forward—GTGATACCACTTCACCTC	255	56	[51]
	Reverse -AGTAAGTGACCAGAATCAG			
*bla*_SHV_	Forward—ACTATCGCCAGCAGGATC	356	53	[51]
	Reverse—ATCGTCCACCATCCACTG			
*bla*_TEM_	Forward—GATCTCAACAGCGGTAAG	786	58	[51]
	Reverse—CAGTGAGGCACCTATCTC			
*bla*_KPC_	Forward—CATTCAAGGGCTTTCTTGCTGC	538	55	[52]
	Reverse—ACGACGGCATAGTCATTTGC			
*bla*_IMP_	Forward—TTGACACTCCATTTACDG	139	55	[52]
	Reverse—GATYGAGAATTAAGCCACYCT			
*bla*_VIM_	Forward—GATGGTGTTTGGTCGCATA	390	55	[52]
	Reverse—CGAATGCGCAGCACCAG			
*bla*_OXA-48_	Forward—GCTTGATCGCCCTCGATT	281	57	[52]
	Reverse—GATTTGCTCCGTGGCCGAAA			
*bla*_NDM-1_	Forward—GGTTTGGCGATCTGGTTTTC	621	52	[53]
	Reverse—CGGAATGGCTCATCACGATC			

## Data Availability

The data are contained within the article.

## Supplementary Materials

The following supporting information can be downloaded at: https://www.mdpi.com/article/10.3390/antibiotics12040653/s1, Table S1. Biochemical analyses of isolates; Table S2. Antibiotic resistance profile; Figure S1. Double disc diffusion test for ESBLs; Figure S2. Modified Hodge test; Figure S3. Detection of ESBL genes (multiplex PCR); Figure S4. Detection of Carbapenemase genes (multiplex PCR).
