# Supplementary material for: Antimicrobial Resistance and β-Lactamase Production in Clinically Significant Gram-Negative Bacteria Isolated from Hospital and Municipal Wastewater
Source: Antibiotics (Basel). 2023 Mar 26;12(4):653. doi: 10.3390/antibiotics12040653 (PMC10135027; doi:10.3390/antibiotics12040653)
Supplement: Supplementary file 1 [file antibiotics-12-00653-s001.zip › antibiotics-2281578-supplementary.pdf]

Supplementary Materials

Antimicrobial Resistance and β-Lactamase Production in Clinically Significant Gram-Negative Bacteria Isolated from Hospital and Municipal Wastewater

Mohammad Irfan *, Alhomidi Almotiri and Zeyad Abdullah AlZeyadi

Department of Clinical Laboratory Sciences, College of Applied Medical Sciences, Shaqra University, AdDawadmi 17464, Saudi Arabia; hsalmutiri@su.edu.sa (A.A.); zalzeyadi@su.edu.sa (Z.A.A.)
* Correspondence: mquraish@su.edu.sa

Table S1. Biochemical analyses of isolates.

Serial No.	Isolates Code	Sample Source	Biochemical Test					Motility
			Indole	Methyl Red	Voges-Proskauer	Citrate	Oxidase test	
1	HI-1	Hospital wastewater	-	-	+	+	-	+
2	HI-2	Hospital wastewater	-	-	+	+	-	-
3	HI-3	Hospital wastewater	-	-	+	+	-	-
4	HI-4	Hospital wastewater	-	-	-	+	-	-
5	HI-5	Hospital wastewater	-	-	-	+	-	-
6	HI-6	Hospital wastewater	-	-	-	+	-	-
7	HI-7	Hospital wastewater	-	-	-	+	+	+
8	MI-1	Municipal wastewater	+	+	-	-	-	+
9	MI-2	Municipal wastewater	+	+	-	-	-	+
10	MI-3	Municipal wastewater	+	+	-	-	-	+
11	MI-4	Municipal wastewater	-	-	+	+	-	-
12	MI-5	Municipal wastewater	-	-	+	+	-	-
13	MI-6	Municipal wastewater	-	-	+	+	-	+
14	MI-7	Municipal wastewater	-	+	-	+	-	+
15	MI-8	Municipal wastewater	-	+	-	+	-	+
16	MI-9	Municipal wastewater	-	+	-	+	-	+
17	MI-10	Municipal wastewater	-	-	-	+	-	-
18	MI-11	Municipal wastewater	-	-	-	+	-	-
19	MI-12	Municipal wastewater	-	-	-	+	+	+
20	MI-13	Municipal wastewater	-	-	-	+	+	+
21	MI-14	Municipal wastewater	-	-	-	+	+	+
22	ME-1	Municipal Treated Water	-	-	+	+	-	+
23	ME-2	Municipal Treated Water	-	-	-	+	+	+

Table S2. Antibiotic resistance profile.

Sample Sources	Sample Types	Isolates Code	Ceftazidime (CAZ)	Cefotaxime (CTX)	Cefepime (FEP)	Imipenem (IMP)	Meropenem (MEP)	Ciprofloxacin (CIP)	Gentamicin (GEN)	Trimethoprim-Sulfameth-oxazole (SXT)	Amoxicillin clavulanate (AMC)	Colistin (CST)
Hospital	wastewater	HI-1	S	R	S	R	R	S	S	R	S	S
		HI-2	S	R	R	R	S	S	S	R	S	S
		HI-3	S	R	S	R	R	R	S	R	R	S
		HI-4	S	R	R	S	S	S	S	S	S	s
		HI-5	S	R	S	S	S	S	R	S	S	s
		HI-6	S	R	R	S	S	S	S	S	S	s
		HI-7	R	S	R	S	S	R	R	S	R	s
Municipal	wastewater	MI-1	S	R	S	S	R	R	S	R	S	S
		MI-2	R	R	S	S	S	R	S	S	S	S
		MI-3	S	R	S	R	S	S	R	S	S	S
		MI-4	S	R	S	R	R	S	S	S	R	S
		MI-5	S	S	S	S	S	S	R	S	S	S
		MI-6	S	R	S	S	S	S	R	S	R	S
		MI-7	R	R	S	S	S	R	S	R	R	S
		MI-8	S	S	R	R	R	S	S	S	R	S
		MI-9	R	R	R	S	R	S	S	R	R	S
		MI-10	S	S	S	R	R	R	S	R	s	s
		MI-11	S	R	R	R	R	S	S	R	R	s
		MI-12	S	S	R	R	R	R	R	S	R	s
		MI-13	S	S	S	R	R	S	S	S	S	s
		MI-14	R	S	S	S	R	R	R	S	S	s
	Treated water	ME-1	S	R	s	S	S	R	R	S	R	S
		ME-2	S	R	S	S	S	S	R	S	S	s

Note: R-Resistant, S-Sensitive.

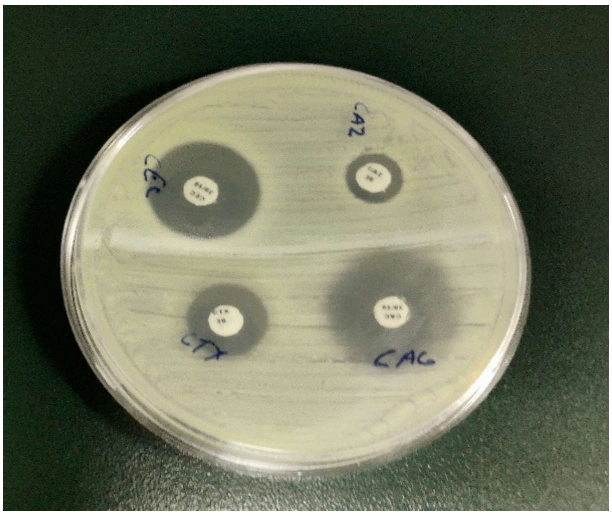

Figure S1. A double disc diffusion test was carried out to verify the presence of ESBL, discs containing ceftazidime (CTX) (30 µg), cefotaxime (CAZ) (30 µg), and a combination of clavulanic acid (30 µg)/10 µg) were used. The isolates were determined to be producers of ESBL if the difference in the zone of inhibition of the drug and inhibitor was at least 5 mm compared to cephalosporin alone..

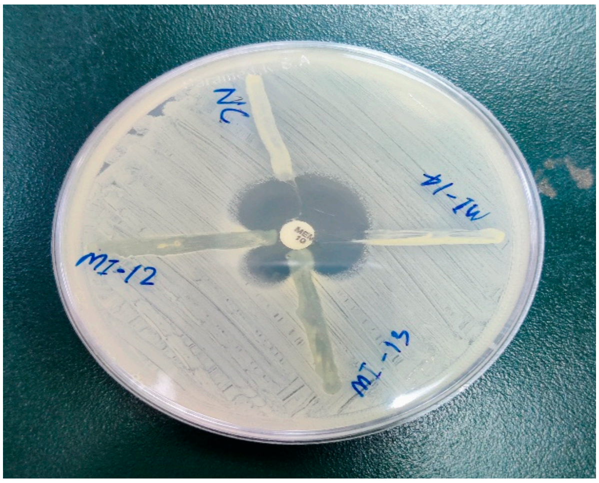

Figure S2. 10 µg of meropenem was left in the middle of the plate and then the isolates were spread in a thin line from the edge of the disc that extended to the edge of the plate. A distorted inhibitory zone in the shape of a clover leaf on the meropenem disc around the growth indicated a positive result.

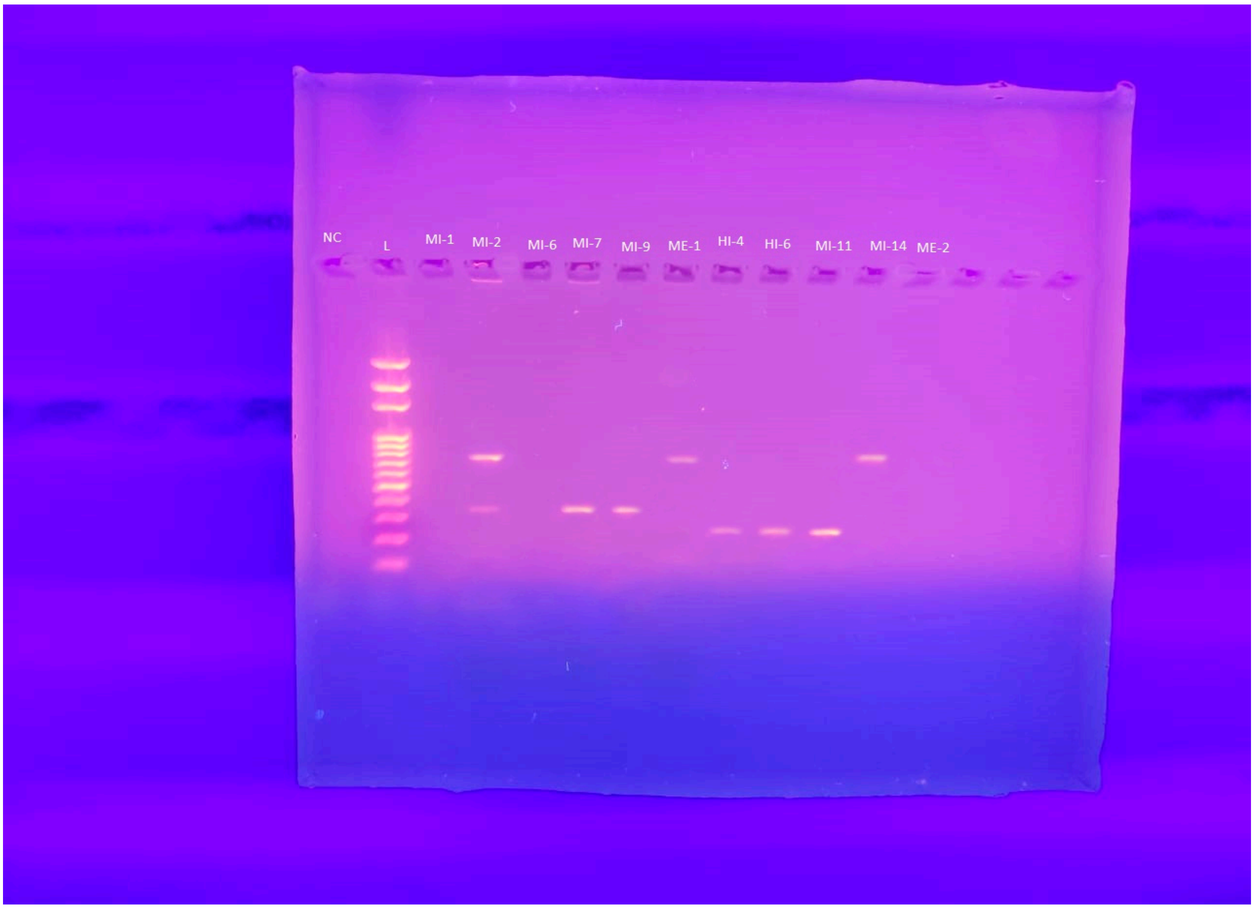

Figure S3. Detection of ESBL genes (multiplex PCR). From left to right: NC – Negative control, L – 100 bp ladder, MI-1, MI-6 and ME-2 – Negative, MI-2- 786 and 356bp bands, MI-7 and MI-9- 356 bp band, ME-1 and MI-14-786 bp band, HI-4, HI-6 and MI-11- 255 bp band.

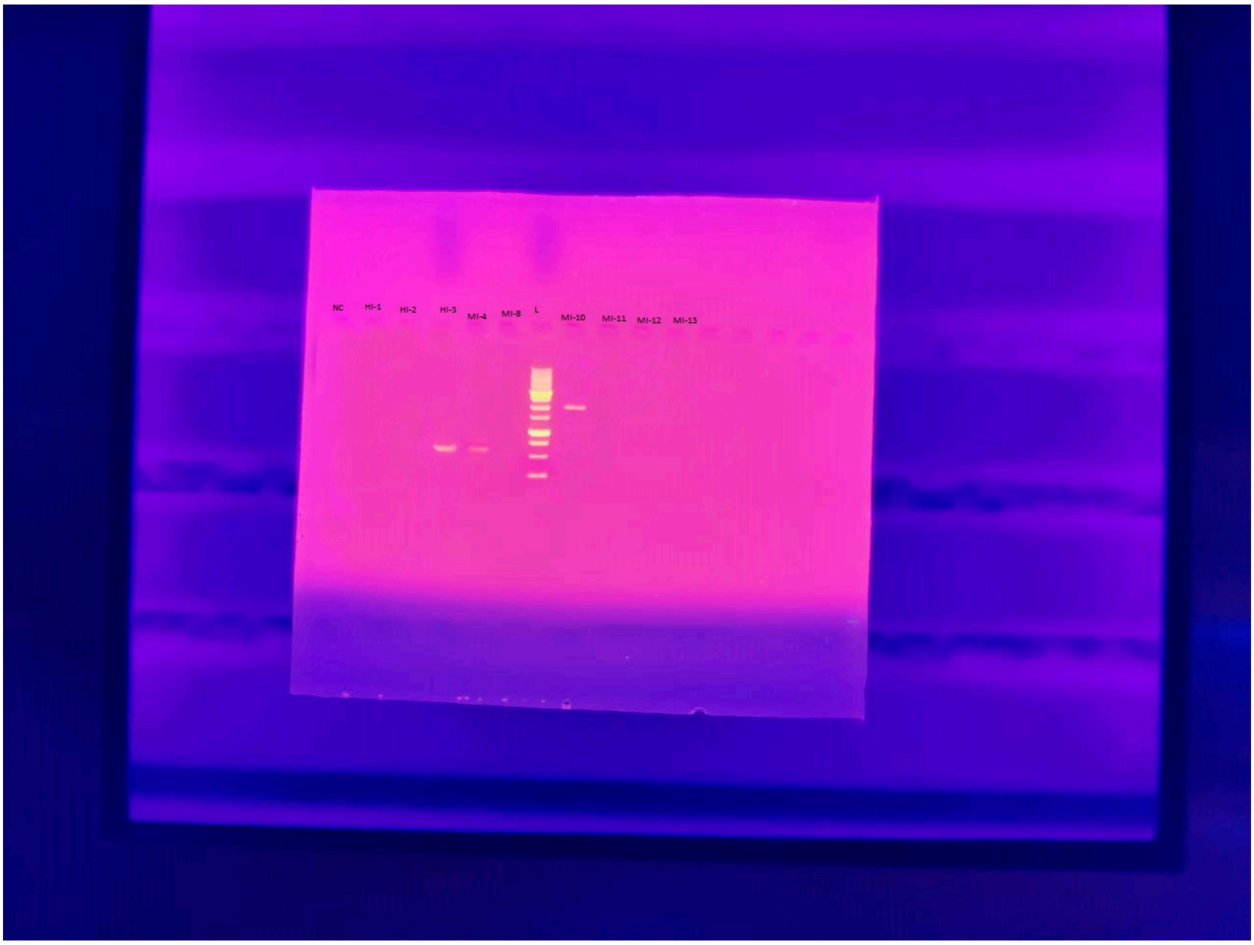

Figure S4. Detection of Carbapenemase genes (multiplex PCR). From left to right: NC – Negative control, HI-1, HI-2, MI-8, MI-11, MI-12, MI-13 are negative. HI-3 and MI-4 - 281 bp band, L – 100 bp ladder, MI-10 - 621 bp band.
